# Status of Thyroid Disorder among the Thyroid Function Test Samples Received in a Laboratory among Postmenopausal Women: A Descriptive Cross-sectional Study

**DOI:** 10.31729/jnma.6191

**Published:** 2021-02-28

**Authors:** Manoranjan Shrestha, Reshmi Shrestha

**Affiliations:** 1 Department of Biochemistry, Nepalese Army Institute of Health Sciences, Bhandarkhal, Kathmandu, Nepal; 2 Department of Pathology, Nepalese Army Institute of Health Sciences Bhandarkhal, Kathmandu, Nepal

**Keywords:** *menopause*, *subclinical hypothyroidism*, *thyroid dysfunction*

## Abstract

**Introduction:**

Thyroid dysfunction prevalence is high in females worldwide which increases with age. Postmenopausal and elderly women are particularly at risk of developing comorbidities and mortality related to thyroid dysfunction. We aimed to study the prevalence of thyroid dysfunction in postmenopausal women in the National Reference Laboratory of Nepal.

**Methods:**

A descriptive cross-sectional study was conducted in National Reference Laboratory from January 2019 to June 2019 including postmenopausal females, ≥49 years. The database of thyroid function test result was used for statistical analysis and proportion of thyroid dysfunction was calculated. The data was collected after approval from the institutional review committee. Statistical Package for Social Sciences version 21 was used to study descriptive data.

**Results:**

Out of a total of 160 postmenopausal females with thyroid function tests, 71 (44.4%) had thyroid dysfunction. Subclinical hypothyroidism was the frequently occurring thyroid dysfunction 51 (32%) followed by subclinical hyperthyroidism 13 (8%), hypothyroidism 3 (2%) and hyperthyroidism 3 (2%). In our study population, thyroid dysfunction peaked at 49 to 58 years of age interval 53 (33.1%) and subclinical hypothyroidism was the most frequent form 38 (23.7%).

**Conclusions:**

Subclinical hypothyroidism was the common thyroid dysfunction in postmenopausal age which peaked at 49 to 58 years of age group. Early postmenopausal females are predisposed to increased risk of comorbidities (cardiovascular disease, osteoporosis with high fracture, depression) which could be exacerbated with thyroid dysfunction; therefore awareness of thyroid dysfunction prevalence and thyroid screening for early management seems appropriate in Nepalese postmenopausal women.

## INTRODUCTION

Thyroid dysfunction is emerging as a global health problem.^1,2^ Many authors have reviewed that the thyroid dysfunction prevalence among the elderly people is rising, especially in the postmenopausal women^3-6^ A study from Nepal^7^ have shown that females >40 years were more prone to develop thyroid dysfunction. In India^8^ the prevalence of thyroid disorders is more common in postmenopausal women.

The diagnosis of thyroid dysfunction is difficult in postmenopausal women since both thyroid and ovarian dysfunction might present with palpitation, insomnia, weight gain etc. The interpretation of the results of thyroid function tests depends on age, comorbidities, and medical treatment which sometimes make the diagnosis of thyroid dysfunction complicated in elderly.^3,4^ Therefore, American association of clinical endocrinology recommends that the older patients, especially women should be screened.^9^

The prevalence of thyroid dysfunction among postmenopausal women has not been addressed so far in Nepal. This study is aimed to find the status of thyroid dysfunction among Nepalese postmenopausal women.

## METHODS

This is a descriptive cross-sectional study conducted in National Reference Laboratory during the period of six months from January 2019 to June 2019. All the data related to females aged 49 years old and above during the study period were included in the study. Ethical approval was obtained from the Institutional Review Committee (Reg no. 252) before the data collection. Females with history of menopause was ≥49 years and wherever clinical data was not available, we considered this age cut off as post menopause based on a recent study^8^ done in Nepal wherein the age for menopause was given to be 48.7 years.

Sample size calculation,


$$\begin{array}{ll} \text{n} & ={\text{Z}}^{\text{2}}\times \text{p}\times \text{q}/{\text{e}}^{\text{2}}\\ & ={\left(1.96\right)}^{2}\times (0.5)\times (1-0.5)/{\left(0.05\right)}^{\text{2}}\\ & =384 \end{array}$$

Where,

n = required sample size
z = 1.96 at 95% Confidence Interval
p = population proportion, 50%
e = margin of error, 5%

The calculated sample size was 384. However, 400 samples received for thyroid function tests were taken into the study.

Thyroid function test results of postmenopausal women were retrieved from the data base. Thyroid function tests included serum measurement of thyroid stimulating hormone (TSH), free triiodothyronine (fT3) and free tetraiodothyronine or thyroxine (fT4). Cases which had only TSH results were excluded from this study. Individuals under treatment for thyroid dysfunction or who had undergone thyroidectomy were also excluded.

Thyroid function tests were done using chemiluminescence immunoassay (CLIA) method (3rd generation immunometric assay technique in fully automated analyzer of Siemens ADVIA chemistry XPT system. Venous blood (3ml) was collected in vacutainer. Serums were separated and fresh sample were run in the automated analyzer on the same day. 800μl of serum was used for the analysis. Tests results were entered in database subsequently. Based on thyroid function test results, patients were categorized into five categories: (1) Euthyroid, (2) Subclinical hypothyroidism, (3) Subclinical hyperthyroidism, (4) Hypothyroidism, and (5) Hyperthyroidism.

Normal range for thyroid function tests given by the manufacturer is shown in Table 1.

**Table 1:** Normal cut off values for thyroid function test.

Thyroid function test	Normal range
Thyroid stimulating hormone (TSH)	0.35 - 5.5 μlU/ml
Triiodothyronine- free (fT3)	2.3 - 1.76 pg/ml
Thyroxine- free (fT4)	0.89 - 1.76 ng/dl

**Table 2:** Criteria for five different categories of thyroid disorder.

Categories of thyroid dysfunction	Criteria
Euthyroid	TSH, fT3 and fT4 values are within normal limit
Subclinical hypothyroidism	TSH- high; fT3 and fT4 values- Normal
Subclinical hyperthyroidism	TSH- Low; fT3 and fT4 values- Normal
Hypothyroidism	TSH- High; fT3 and fT4 values -Low
Hyperthyroidism	TSH- low; fT3 and fT4 values-High

For statistical analysis, SPSS 21 software was used. Data are presented in the form of frequency, which is expressed as percentage and means with standard error.

## RESULTS

**Figure 1.**
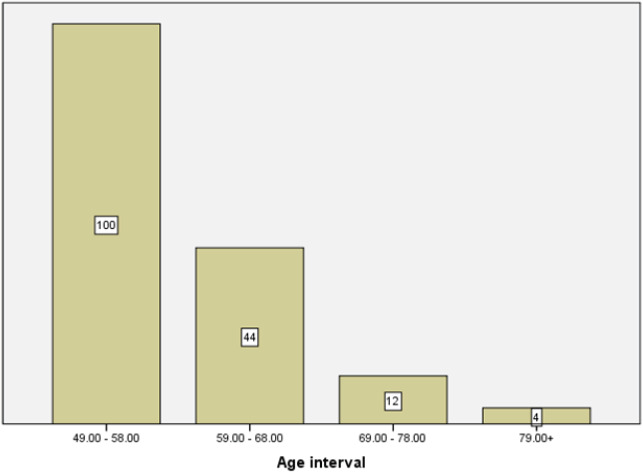
Age distribution of postmenopausal women.

Of total 400 females who had thyroid function tests, 160 females were found to be in the postmenopausal age group. The mean age of postmenopausal females was 57.9 years, ranging from 49 to 82 years of age. The age distribution of postmenopausal women is shown in Figure 1. Maximum number of cases was seen in the age group of 49 to 58 years.

**Figure 2.**
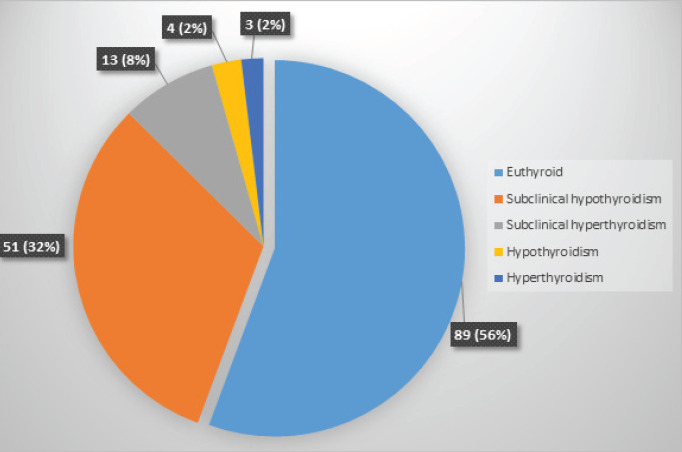
Frequencies of different categories of thyroid dysfunction.

Of total 160 cases, thyroid dysfunction was observed in 71 (44.4%) and the rest were euthyroid 89 (57.6%). The frequencies of euthyroid and different categories of thyroid dysfunction are presented in Figure 2. Out of the 71 cases of thyroid dysfunction, subclinical hypothyroidism was the most frequent thyroid dysfunction in postmenopausal women 51 (71.8%).

Mean of TSH, fT3 and fT4 test results in different age groups are depicted in Table 3.

**Table 3:** Mean value of Thyroid function test results at different age intervals in postmenopausal women.

Thyroid function tests	Age interval			
	49-58 years	59-68 years	69-78 years	≥79 years
TSH (mean ±S.E^*^)	5.17±0.50	4.17±0.72	3.34±0.66	35.92±33.6
T3 (mean±S.E^*^)	3.05±0.06	2.82±0.05	2.99±0.14	2.4±0.37
T4 (mean±S.E^*^)	1.17±0.02	1.19±0.38	1.23±0.07	0.9±0.14

* Standard error of mean

**Figure 3.**
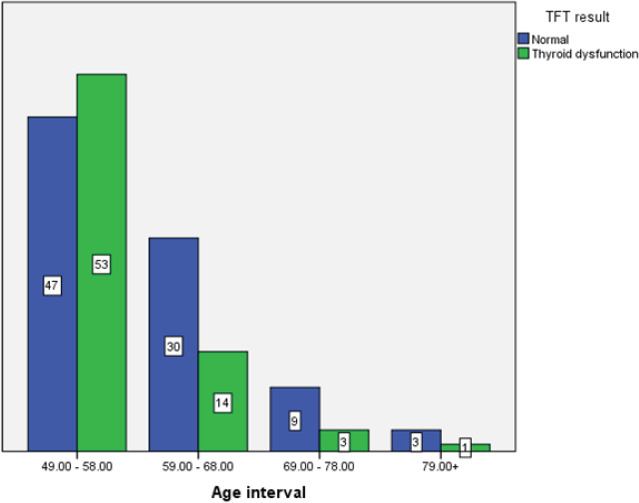
Distribution of euthyroid and thyroid dysfunction at different age intervals.

Distribution of thyroid dysfunction and euthyroid state across the different age intervals is presented in Figure 3. The cases with thyroid dysfunction was highest in the age range of 49-58 years, exceeding than that of euthyroid population (53% of thyroid dysfunction vs 47% of euthyroid state out of 100 cases in the age group of 49-58 years).

**Figure 4.**
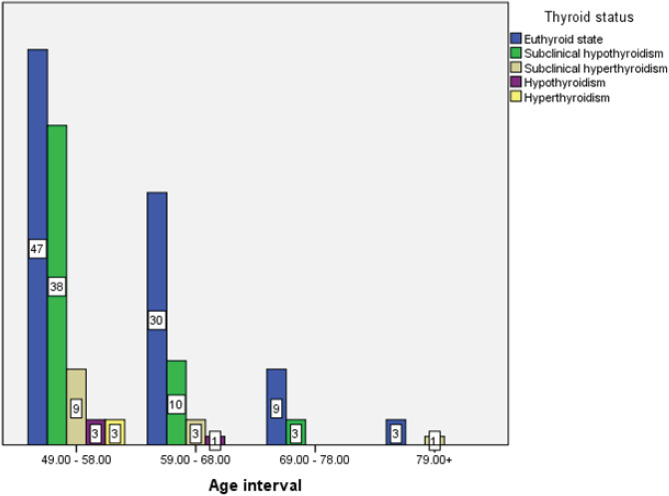
Distribution of different categories of thyroid dysfunction at different age groups.

Subclinical hypothyroidism which was the frequently occurring thyroid dysfunction peaked at the age group of 49 to 58 years as demonstrated in Figure 4.

## DISCUSSION

Thyroid diseases predominantly affect females worldwide and are highest among postmenopausal and elderly women.^3,10^ Risk of osteoporosis and cardiovascular disease increases in postmenopausal women and it could also be associated with thyroid dysfunction (TD). Subclinical hypothyroidism (SCHO) is associated with impaired left ventricular diastolic function at rest, systolic dysfunction on effort, and enhanced risk for atherosclerosis and myocardial infarction which could be reversed with levothyroxine replacement.^5^ Therefore, clinician should be aware of and rule out thyroid dysfunction in postmenopausal women. We attempted to study the prevalence of thyroid dysfunction particularly in Nepalese postmenopausal women who are not only at risk of facing postmenopausal symptoms but might have associated thyroid dysfunction with increased cardiovascular risk, cognitive dysfunction, osteoporosis etc.

Several studies conducted in different parts of Nepal have revealed overall higher prevalence of thyroid dysfunction in females when compared to males with male to female ratio ranging from 4:1 to 8:^1,5,11-13^ Aryal et al^14^ and Dangol et al^7^ have demonstrated 24% of thyroid dysfunction in Nepalese female population of Kavre and Tansen region (a hilly Mid-West Nepal). Baral et al^15^ presented 31% of prevalence of thyroid dysfunction among females in Terai. Jayan et al,^11^ Khatiwada et al^16^ and Yadav et al^17^ however have found a higher prevalence of 42% among female population in South Western Nepal and 40% in BPKIHS, Dharan and far Western Nepal respectively. In contrast, one of the study^18^ in Far Western region of Nepal has revealed only 15% of thyroid dysfunction in females. In Indian population, an epidemiological study has reported higher prevalence of TD in females however lesser frequency than in Nepal accounting to 11.4%. Studies of prevalence in postmenopausal women conducted by Bordoloi et al^8^ and Deshmukh et al^19^ from India and Niafar et al^20^ from Iran have reported 13%, 20% and 12.7% respectively. A review by Domingo et al^21^ have shown high prevalence of TD in elderly females than in males (16% vs 4%) which was based on Canadian community health survey, 2005. In contrast, we found 44.4% of thyroid dysfunction among postmenopausal women in our study. The higher prevalence in our study in contrast to the Western world could be due to higher frequency of iodine deficiency in the population of pre-era of iodization.

We found subclinical hypothyroidism as the most frequent thyroid dysfunction in postmenopausal women in consistent with Bordoiloi et al^8^ (13%); Desmukh et al^19^ (20%); Domingo et al^21^, Niafar et al^21^ (5.8%); Wilson et al.^22^ Studies conducted in Nepal have also reported increased prevalence of subclinical hypothyroidism in females ranging from 10% to 30%.^12,16,24^ We observed higher proportion of subclinical hypothyroidism in postmenopausal age accounting to 32%. Higher prevalence of subclinical hypothyroidism has been associated with iodine deficiency primarily in Himalayan belt and hilly region due to soil erosion in Nepal. In contrast, prevalence of common thyroid dysfunction in Western region of Nepal has been reported as hyperthyroidism by Baral et al^15^ and Yadav et al.^24^ It is attributed possibly due to thyroid autonomy of endemic goiter (toxic nodular goiter or toxic adenoma) or use of excess iodized salt in these regions.

Large population based survey^23^ on thyroid dysfunction and several reviews^2,10^ have stated that its frequency increases with advancing age. Canadian community health survey, 2005 have demonstrated that advancing age is associated with increased frequency of thyroid dysfunction as shown by the Canadian statistics. TD was 9% in the age group of 45-64 years; 14% at 65 to 84 years and 16% in the age group ≥85 years.^23^ In the context of Nepal, most of the studies, however have presented increased frequency of TD either in the form of either hyperthyroidism or SCHO among active reproductive age group between 21 to 40 years or 31 to 45 years.^15,11,17,24^ which is argued to be due to high prevalence of toxic nodular goiter or iodine deficiency. One of the studies from Tansen^7^ however demonstrated the significant increase in thyroid dysfunction in females above 40 years. In our study, among postmenopausal women, highest proportion of thyroid dysfunction (predominantly, SCHO) was found in the age group of 49 to 58 years.

Prevalence of thyroid dysfunction is related to dietary iodine intake, geographical variation, genetic predisposition, ethnic group, prevalence of antithyroid antibodies and drug intake that affect thyroid hormone especially in elderly. In iodine sufficient areas, increased TSH level is observed with ageing and also is associated with various diseases such as autoimmunity dysfunction symptoms in the elderly or it could be masked by other comorbidities. One of the study^24^ has highlighted on the lack of awareness of menopausal health problems in Nepalese women which could be the reason for decrease visit to the doctor and regular health checkup.

This study would have been more helpful to identify prevalent pattern in general population if we could have a larger sample size with greater detail clinical history, other laboratory tests such as evaluation of urinary iodine to determine iodine deficient status or excess status and anti TPO antibodies study to find out the cause of thyroid usually associated with high titer of autoantibodies, type II diabetes, gastrointestinal disorders and drug induced thyroid dysfunction whereas in iodine deficient population usually the cause is toxic multinodular goiter and toxic adenoma.^12^ Decreased prevalence in the elderly women in our study could be because of small sampling in that age group which could be explained by the lack of awareness of thyroid dysfunction. However, it highlights the further need of exploring thyroid function status in the postmenopausal and elderly women which would provide us insight to the burden of disease and application of preventive measures at the earliest.

## CONCLUSIONS

Postmenopausal women are more vulnerable to develop subclinical hypothyroidism in our population and are therefore at higher risk of progressing into overt hypothyroidism and developing cardiovascular disease, cognitive impairment, depression and osteoporosis with increased fracture risk. Clinician should be aware of the thyroid dysfunction in this subset of female population and test for serum TSH and thyroxine should be done whenever suspicious.
